# 67-kDa Laminin Receptor-Mediated Cellular Sensing System of Green Tea Polyphenol EGCG and Functional Food Pairing

**DOI:** 10.3390/molecules27165130

**Published:** 2022-08-11

**Authors:** Yoshinori Fujimura, Motofumi Kumazoe, Hirofumi Tachibana

**Affiliations:** Division of Applied Biological Chemistry, Department of Bioscience and Biotechnology, Faculty of Agriculture, Kyushu University, Fukuoka 819-0395, Japan

**Keywords:** green tea, catechin, EGCG, 67LR, cGMP, food factor sensing, functional food pairing

## Abstract

The body is equipped with a “food factor-sensing system” that senses food factors, such as polyphenols, sulfur-containing compounds, and vitamins, taken into the body, and plays an essential role in manifesting their physiological effects. For example, (−)-epigallocatechin-3-*O*-gallate (EGCG), the representative catechin in green tea (*Camellia sinensi* L.), exerts various effects, including anti-cancer, anti-inflammatory, and anti-allergic effects, when sensed by the cell surficial protein 67-kDa laminin receptor (67LR). Here, we focus on three representative effects of EGCG and provide their specific signaling mechanisms, the 67LR-mediated EGCG-sensing systems. Various components present in foods, such as eriodictyol, hesperetin, sulfide, vitamin A, and fatty acids, have been found to act on the food factor-sensing system and affect the functionality of other foods/food factors, such as green tea extract, EGCG, or its *O*-methylated derivative at different experimental levels, i.e., in vitro, animal models, and/or clinical trials. These phenomena are observed by increasing or decreasing the activity or expression of EGCG-sensing-related molecules. Such functional interaction between food factors is called “functional food pairing”. In this review, we introduce examples of functional food pairings using EGCG.

## 1. Introduction

**Figure 1:**
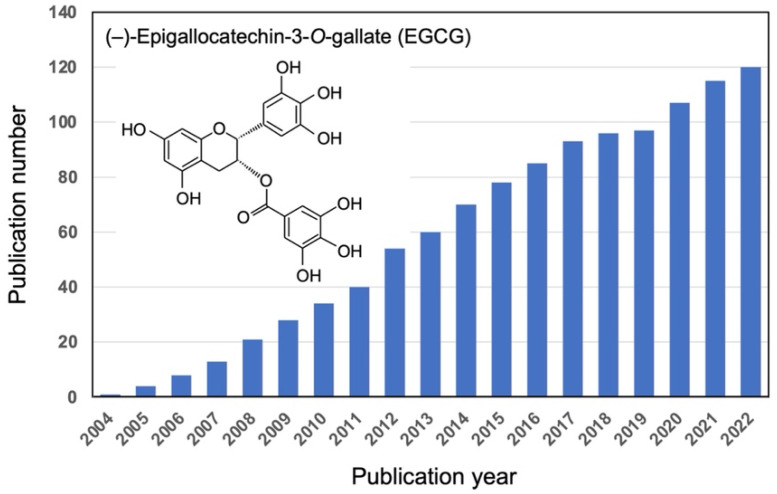
Trends in research on biological and pharmacological activities of EGCG via 67LR. This figure shows the results of a PubMed search for articles/reviews published between 2004 and 2022 using the keywords “laminin receptor” and “EGCG”.

Food factors are known to be food components, mainly non-nutrient components that are expected to have physiological effects, such as phytochemicals. Several studies have investigated the functionality of individual food factors; however, not much is known about the functionality of multiple food factors consumed simultaneously. Additionally, the body has a mechanism for sensing ingested food factors; this system and the food factors consumed simultaneously are essential in the expression of physiological functions of a specific dietary compound. For example, vitamin A manifests its effects when sensed by the retinoic acid receptor (RAR). (−)-Epigallocatechin-3-*O*-gallate (EGCG), the major green tea catechin, exerts various pharmacological effects, including anti-cancer, anti-inflammatory, and anti-allergic effects, when sensed by the 67-kDa laminin receptor (67LR) [1]. Activation of RAR by vitamin A enhances the physiological effects of EGCG by increasing the expression of the EGCG-sensing receptor 67LR [2]. A PubMed search for “laminin receptor” and “EGCG” showed that more than 100 articles have been published since 2004 (Figure 1). Various physiological effects and 67LR-mediated cellular sensing systems of EGCG have been revealed. The bioactivity of EGCG is regulated by modulating the activity or expression of molecules on the EGCG signaling pathways, i.e., EGCG-sensing-related molecules. The anti-cancer effects of EGCG via 67LR have been shown to differ between cancer types (e.g., melanoma vs. multiple myeloma (MM)) and their specific EGCG-sensing pathways [3,4]. 67LR is also essential in manifesting the anti-inflammatory effect of EGCG through attenuating Toll-like receptor 4 (TLR4) signaling [5], and a potent target for relieving inflammation caused by obesity [6]. If food factors can enhance the activity or expression of such EGCG-sensing-related molecules, they can present a useful combined effect with EGCG. We also searched for food factors that enhance EGCG sensing. Flavanones and citrus polyphenols enhance EGCG signaling, thereby potentiating its effects and those of green tea: anti-obesity [7], muscle atrophy prevention [8], anti-cancer [9], and anti-allergic [10] effects. Such a functional interaction between food factors is called “functional food pairing”. Epidemiological study has shown functional interactions between citrus and green tea [11], and we recently demonstrated the anti-obesity effects of the combined intake of α-glucosyl hesperidin and green tea in a human interventional study [12].

Generally, the intensity of the functional effect of a food factor is believed to be determined by the amount of food consumed. However, this review focuses on topics showing that the bioactivities of a food factor are greatly influenced by other food factors consumed simultaneously. Understanding such functional food pairings will help overcome the problems of using EGCG/green tea alone; i.e., non-specific/harmful effects based on high concentration use and low efficacy, and may lead to providing new strategies to better utilize the diverse bioactivities of EGCG/green tea. Here, we describe the functional food pairings of EGCG with various food factors while unraveling the molecular basis of the EGCG-sensing system.

## 2. Green Tea Catechin EGCG-Sensing Receptor

### 2.1. Identification of the Green Tea Polyphenol EGCG-Sensing Receptor

The representative components responsible for the bioactivity of green tea are catechins, a subgroup of polyphenols. These catechins include EGCG, (−)-epigallocatechin (EGC), (−)-epicatechin-3-gallate (ECG), and (−)-epicatechin (EC). Their C-2 epimeric isomers are (−)-catechin (C), (−)-gallocatechin (GC), (−)-catechin-3-*O*-gallate (CG), and (−)-gallocatechin-3-*O*-gallate (GCG). The non-epicatechins (GCG, CG, GC, and C) in tea leaves are known to be less abundant than the epicatechins (EGCG, ECG, EGC, and EC). These four epicatechins are readily epimerized by heat treatment and pH conditions to produce the corresponding non-epimeric forms [13]. EGCG is found only in tea leaves from *Camellia sinensis*, and is therefore a characteristic component of green tea. It is the most abundant of the catechins and shows the strongest bioactivity among catechins. Pharmacokinetic studies in human subjects have indicated that the maximum plasma concentration (C_max_) after a dose of EGCG in our daily life is usually <1.0 μM. On the other hand, oral administration of an EGCG supplement or a standardized green tea extract Polyphenon E^TM^, containing 60% EGCG, has also shown that the plasma level of EGCG can reach as high as 7 μM [14]. Most pharmacological effects of EGCG observed in vitro experiment and cell-free systems have been obtained at concentrations (10–100 μM) considerably higher than those reported in vivo after intake of green tea or EGCG. Furthermore, the intracellular levels of EGCG were much lower than the extracellular ones. Identifying the proteins that EGCG exhibits high affinity towards is the first step toward deciphering the molecular mechanisms underlying its anti-cancer effect. Several such proteins have been identified using in vitro techniques [15,16]. All of them are essential to the anti-cancer effect of EGCG in cancer cells. However, for this effect to be observed, EGCG concentrations higher than the dissociation constant (*K_d_*) values are required.

**Figure 2:**
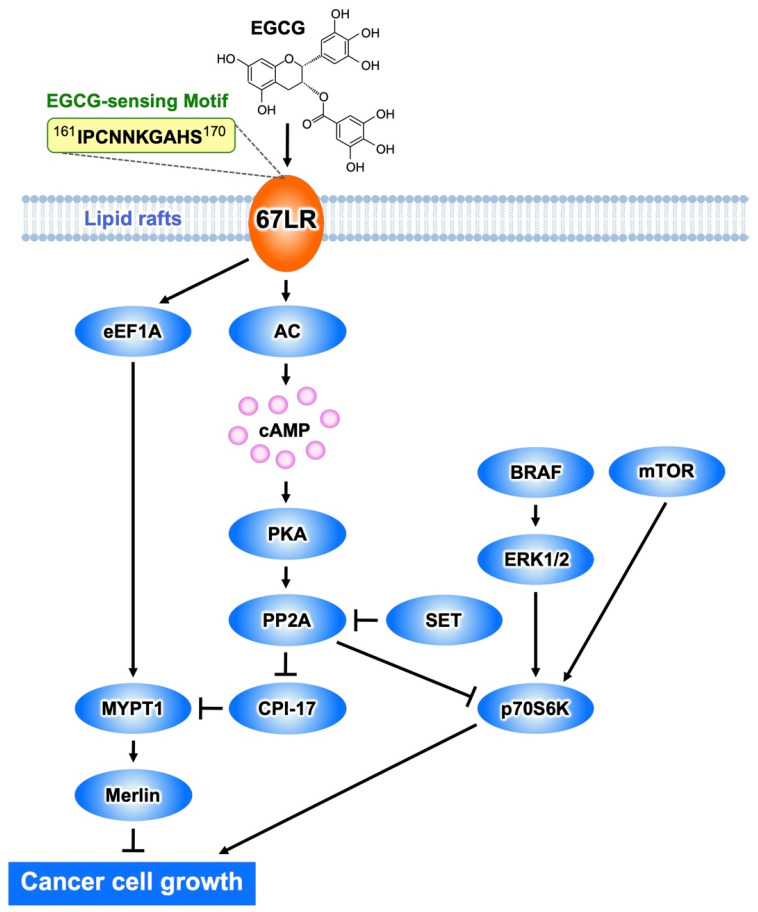
The EGCG-sensing pathway inhibits melanoma cell growth and proliferation by a mechanism mediated by 67LR. The interaction EGCG−67LR results in the activation of MYPT1 via AC/cAMP/PP2A- or the eEF1A-dependent pathway. Consequently, merlin is activated, and dephosphorylates MRLC. BRAF/ERK or mTOR-mediated activation of p70S6K is inhibited by PP2A.

All-trans-retinoic acid (ATRA) amplifies the attachment of EGCG to the cancer cell surface. This molecular interaction was evaluated by using a surface plasmon resonance binding analysis. cDNA libraries were obtained from cancer cells either treated with ATRA or were untreated. Subtractive cloning was used to identify the differences in the composition of mRNA transcripts of the two types of cells. Thus, 67LR was identified as an isolated single target for EGCG and found to be essential to the EGCG binding to the cancer cell surface and, thus, to its inhibitory effect on cell growth at the concentration of 5 μM [1]. The EGCG binding site is located on the extracellular domain of 67LR, corresponding to the 161–170 region [17] (Figure 2). The calculated *K_d_* value for the EGCG−67LR complex is about 40 nM. Most 67LR proteins are located in the region of lipid rafts, plasma membrane microdomains enriched in cholesterol and sphingolipids, rather than the non-lipid rafts [18]. This type of distribution matches the plasma membrane-associated EGCG concentration determined after treating cells with EGCG [19].

In vivo studies confirmed that 67LR functions as a receptor of EGCG, which is critical to its anti-cancer effect. Thus, EGCG treatment substantially inhibited tumor growth in mice implanted with B16 cells harboring a control short hairpin RNA (shRNA), but not in those implanted with 67LR-ablated B16 cells [20].

Furthermore, 67LR is associated with adherence of tumor cells, migration, invasion, and metastasis [21]. It is also a target molecule for pathogenic prion proteins [22], Sindbis virus [23], and dengue virus [24]. Caffeine and other catechins (C, EC, EGC, and quercetin) do not bind to the surface of 67LR-expressing cells nor affect their growth at the concentration of 5 μM [1]. Galloylated, but not non-galloylated, catechins attach to the cancer cells. The binding ability of pyrogallol-type catechins (EGCG and GCG) is higher than that of catechol-type catechins (ECG and CG) [25], in correlation with their biological activities. Downregulation of the expression of 67LR reduces the activity of galloylated catechins. Thus, both the galloyl moiety and B-ring hydroxylation may be involved in biological activities of these compounds mediated by 67LR.

Strictinin, one of esters of gallic acid with a polyol, is a hydrolysable ellagitannin in green tea. It suppresses interleukin (IL)-4 signaling [26] by interacting with the non-lipid raft-associated IL-4 receptor (IL-4R) on the surface of the cell membrane [27]. Downregulation of 67LR does not interfere with this effect. The *K_d_* value of IL-4R-strictinin complex is 4.53 μM, whereas that of complex of IL-4R with EGCG, which is mostly located in the lipid-raft region, is about 155 μM [27]. Thus, both the flavan-3-ol structure and galloyl moiety might play a crucial role in the interaction between 67LR and EGCG.

Recently, we revealed that EGCG formed oligomers by binding to 67LR [28]. Oligomer formation occurs after absorption, possibly in a manner similar to that of highly oligomerized procyanidin. Our data indicate a possible mechanism underlying the strong in vivo biological activities of EGCG.

Procyanidins are one of the well-known bioactive polyphenols in grape seeds and apple peels. Recent studies showed that these compounds have anti-cancer properties [29,30]. For instance, procyanidin C1 (PC1), an EC trimer, has anti-cancer effects [30]; however, its molecular mechanisms underlying this effect remain unknown. Recently, we revealed that PC1 also binds to 67LR with a *K_d_* value of 2.8 µM [31]. This interaction results in the activation of protein kinase A (PKA) and protein phosphatase 2A (PP2A). Consequently, it inhibits the growth of melanoma cells, by downregulation of phosphorylation levels of the C-kinase potentiated protein phosphatase-1 inhibitor protein of 17-kDa (CPI17) and dephosphorylation of myosin regulatory light chain (MRLC) proteins, followed by actin cytoskeleton remodeling. In the same study, catechin dimers procyanidin B1 (PB1) and procyanidin B2 (PB2) showed lack of cell surface binding with no significant activity on phosphorylation levels of MRLC in melanoma cells. Taken together, PC1 has anti-melanoma effects mediated by a 67LR-dependent mechanism. The mechanisms by which other EC oligomers interact with 67LR, such as tetramers and pentamers, remain unknown and further studies are needed.

### 2.2. Inhibitory Actions of EGCG on Cancer Cell Growth Mediated by 67LR

Melanoma is the deadliest skin cancer and is notorious for its resistance to treatment, but recent clinical trials of targeted therapies have shown promise [32,33]. On the other hand, many anti-cancer drugs are natural products or their derivatives, pointing to the usefulness of natural products in drug discovery. Functional genetic screening identified eukaryotic elongation factor 1A (eEF1A) as a critical anti-proliferative factor for melanoma cells [20]. eEF1A regulates various cellular processes, including the translation process in eukaryotes. EGCG substantially inhibited tumor growth in mice inoculated with mouse melanoma cells harboring a control shRNA, but not in mice inoculated with eEF1A-ablated melanoma cells, highlighting the role of eEF1A plays in the EGCG-induced anti-cancer effect.

We mentioned above that EGCG binding to 67LR results in cell growth inhibition as a result of a suppressing effect on the phosphorylation of MRLC at Thr18/Ser19 [34]. This is determined by EGCG-induced downregulation of the phosphorylation level of the myosin phosphatase target subunit 1 (MYPT1) at Thr696. Consequently, myosin phosphatase activity is upregulated.

EGCG administration substantially inhibited tumor volume increase in mice inoculated with B16 cells harboring a control shRNA, but not in mice inoculated with MYPT1-knockdowned B16 cells, indicating that MYPT1 is also an essential element for EGCG-induced cancer prevention. In eEF1A- or 67LR-knockdowned mouse melanoma cells, EGCG does not induce downregulation of the phosphorylation level of MYPT1^Thr696^ at the concentration of 1 μM. These findings indicate that MYPT1 plays a crucial role in EGCG signaling from both 67LR and eEF1A (Figure 2).

Abnormal activation of BRAF is the most frequently observed mutation event in patients with melanoma and is associated with constitutive hyperproliferation. Selective BRAF inhibition improves the outcomes of patients with mutated BRAF melanoma. Unfortunately, acquired resistance to BRAF inhibition is the problem. Therefore, there is an urgent need to identify new strategies of treatment of melanoma. EGCG (5 μM) inhibits melanoma cell growth independent of BRAF inhibitor sensitivity by regulating the activity of PP2A. Functional genetic screening identified this enzyme as a key player in lowering the growth of melanoma cells [3]. EGCG binding to 67LR elicits activation of PP2A via the adenylate cyclase (AC)/cyclic adenosine monophosphate (cAMP) axis, causing melanoma-specific mammalian target of rapamycin (mTOR) inhibition and merlin activation related to tumor suppression (Figure 2). Furthermore, the inhibition of mTOR causes strong synergism with the BRAF inhibitor in BRAF inhibitor-resistant melanoma. Additionally, Suvar3–9, an enhancer-of-zeste trithorax (SET), an oncoprotein that inhibits the activity of PP2A, is overexpressed in melanoma. EGCG (5 μM) activates PP2A and inhibits cell growth without affecting SET expression in melanoma. SET silencing significantly enhances 67LR/PP2A signaling in vivo and, consequently, the EGCG-elicited anti-melanoma activity [35]. Therefore, SET is expected to be an important target molecule for EGCG to potentiate its anti-melanoma action.

### 2.3. Cancer Cell Killing Effects of EGCG Mediated by 67LR

Green tea consumption has shown beneficial effects in patients with oral cancer [36], colorectal adenomas [37], prostate cancer [38], and early-stage chronic lymphocytic leukemia [39]. In a Phase II clinical trial in patients with chronic lymphocytic leukemia, Polyphenon E^TM^, containing 60% EGCG, the first botanical drug approved by the US Food and Drug Administration for the treatment of patients with external genital and perianal warts, showed a strong anti-cancer effect without causing serious side effects [39].

EGCG (5 or 10 μM) induces growth arrest and apoptosis in multiple myeloma (MM) [40] and acute myeloid leukemia (AML) cells [41] derived from cell lines and human patients; however, it has no impact on the growth of normal cells such as normal human peripheral blood mononuclear cells (PBMCs). The levels of 67LR expression are strongly upregulated in myeloma cells compared with PBMCs. The specific pro-apoptotic effect of EGCG on MM cells is prevented by RNAi-mediated inhibition of 67LR expression, further highlighting the importance of this receptor in the cancer killing effect of EGCG.

Changes in the clustering of lipid rafts activate various cellular signaling pathways, including those involved in apoptosis. EGCG (5 μM), but not biologically inactive EC (10 μM), induced clustering of lipid rafts in MM cells [42]. An anti-67LR antibody treatment blocked EGCG-elicited clustering of lipid rafts, whereas control antibody treatment did not. Lipid rafts are microdomains enriched in sphingolipids and cholesterol, and changes in their quantity cause changes in the integrity and function of lipid rafts. Cholesterol pretreatment impeded EGCG-induced lipid raft clustering and apoptosis in MM cells, indicating that lipid raft clustering is indispensable for the apoptotic cell death induced by EGCG.

**Figure 3:**
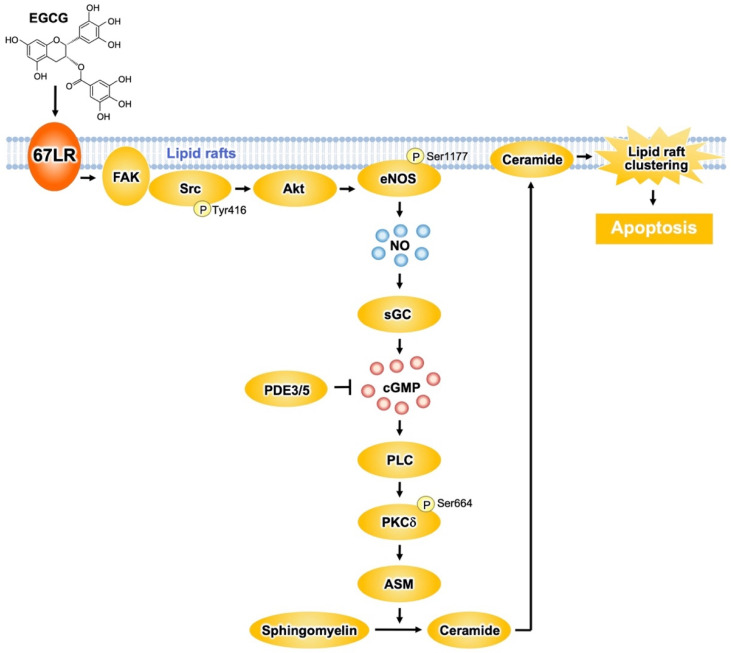
The 67LR-dependent EGCG-sensing pathway involved in multiple myeloma (MM) cell death. EGCG induces apoptosis in multiple myeloma (MM) cells via the activation of the 67LR/ FAK/Src/Akt/eNOS/NO/sGC/cGMP/ PLC/PKCδ/ASM signaling pathway.

Acid sphingomyelinase (ASM) has an essential role in lipid raft clustering through the production of ceramide. The expression of ASM is drastically upregulated in myeloid cells compared to its normal counterpart [3]. EGCG (5 μM) induced ASM activation in myeloid cells, but did not affect its normal counterpart [42]. Furthermore, an anti-67LR antibody treatment neutralized EGCG-elicited ASM activation, indicating that 67LR acts as a critical player in the effect of EGCG. Therefore, EGCG activates the sphingolipid cascade by eliciting the ASM/67LR axis, and this enzyme is indispensable for EGCG-elicited apoptosis by clustering of lipid rafts in MM cells (Figure 3).

Protein kinase Cδ (PKCδ) plays an essential role in the apoptosis-inducing pathway. EGCG (1 μM) upregulated the phosphorylation levels of PKCδ^Ser664^ in myeloid cells, without affecting its normal counterpart [42]. Pretreatment of the cells with an anti-67LR antibody inhibits the elevation of phosphorylation levels of PKCδ^Ser664^ induced by EGCG. Moreover, pharmacological inhibition of PKCδ neutralized the EGCG-induced ASM activation. Knockdown of ASM in myeloma cells had no influence on EGCG-elicited upregulation of phosphorylation levels of PKCδ^Ser664^. Thus, ASM activation elicited by EGCG may be a subordinate event that is triggered after upregulation of PKCδ activity. Oral intake of EGCG induced the activation of caspase 3, the determining step of apoptosis signaling, in myeloid cells. In addition, EGCG upregulated the phosphorylation levels of PKCδ^Ser664^ and increased ASM activity in cancer cells, suggesting that EGCG elicits the PKCδ/ASM axis in myeloid cells in vivo [42] (Figure 3).

67LR contributes to shear stress-elicited expression of nitric oxide synthase 3 (also endothelial nitric oxide synthase (eNOS)) [43]. EGCG (5 μM) increased NO levels in myeloid cells, but not in PBMCs from healthy donors [4]. This increase results from eNOS activation owing to phosphorylation at Ser1177 by Akt. Anti-67LR antibody pretreatment in myeloid cells inhibited eNOS^Ser1177^ phosphorylation and prevented the increase in Akt kinase activity induced by EGCG (5 μM) [4]. Therefore, EGCG elicits the upregulation of NO levels by activating the 67LR/Akt/eNOS axis (Figure 3).

NO upregulates the levels of cyclic guanosine monophosphate (cGMP) via soluble guanylate cyclase (sGC)-dependent mechanisms. EGCG (5 μM) upregulates cGMP levels in myeloid cells without affecting its normal counterpart [4]. Anti-67LR antibody or sGC inhibitor pretreatment prevented EGCG-induced cGMP elevation. The pharmacological inhibition of sGC also attenuated EGCG-induced apoptosis and activation of ASM. Altogether, these data indicate that activation of the 67LR/cGMP signaling axis is the critical event for EGCG-induced apoptosis (Figure 3). Other catechins did not have any influence on cGMP levels.

EGCG elicits apoptotic cell death in several types of malignant cells though the induction of cGMP. However, the upstream mechanisms that occur after cGMP induction are not completely understood. We demonstrated that a cGMP increase determined by EGCG induced PKCδ^Ser664^ phosphorylation and, consequently, ASM activation [44]. EGCG upregulated phospholipase C (PLC) activity in a similar manner to the treatment with cGMP inducers. PLC is involved in the production of diacylglycerol (DAG), which is required for PKCδ activation. Pharmacological inhibition of PLC prevents EGCG-induced ASM activation. Pharmacological inhibition of DAG strongly increases the activity of EGCG. Collectively, these findings indicate that EGCG induces apoptotic cell death in MM cells by activating the cGMP/PLC/PKCδ/ASM signaling pathway [44] (Figure 3).

The proto-oncogene c-Src greatly contributes to the anti-cancer effect of EGCG [45]. EGCG promotes c-Src^Tyr416^ phosphorylation, which is essential for the activity of the enzyme. Focal adhesion kinase (FAK), a regulator of Src phosphorylation, is colocalized with 67LR, and EGCG enhances the interaction between FAK and 67LR. Furthermore, pharmacological inhibition of Src and FAK prevents the early mechanisms in EGCG-elicited apoptosis: increase of Akt activity, upregulation of cGMP levels, and enhance of ASM activity. These observations suggest that FAK/Src highly contributes to the upstream signaling of EGCG-induced Akt/cGMP/ASM activation (Figure 3).

### 2.4. Anti-Inflammatory and Anti-Allergic Actions of EGCG Mediated by 67LR

**Figure 4:**
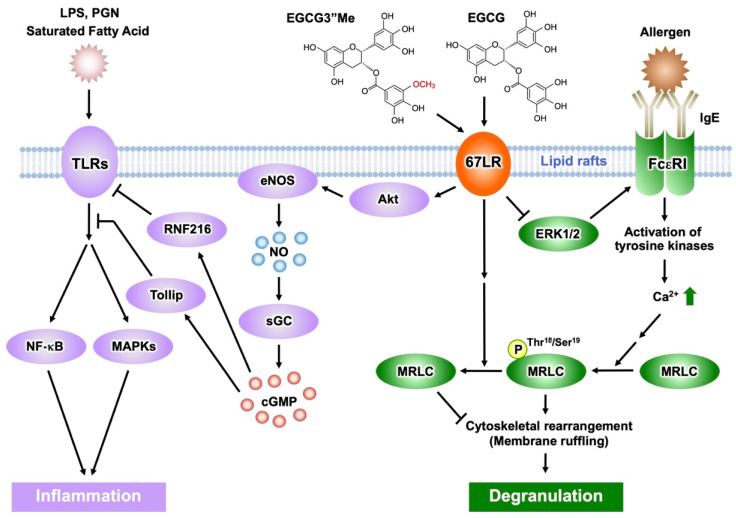
Activation of EGCG-sensing pathway following its binding to 67LR results in anti-inflammatory and anti-allergic actions. The anti-inflammatory effect of EGCG and EGCG3″Me results from inhibition of TLR signaling mediated by 67LR, Tollip, and RNF216. EGCG/EGCG3″Me binding to 67LR suppresses MRLC phosphorylation and the histamine release from basophils. EGCG/EGCG3″Me also inhibits ERK1/2 phosphorylation, reducing FcεRI expression.

Lipopolysaccharide (LPS) is one of the major components of Gram-negative bacteria, is a ligand of Toll-like receptor 4 (TLR4) signaling, and an inducer of inflammatory cytokines, which are involved in the onset and pathological development of septic shock. EGCG prevents LPS-elicited sepsis in mice and suppresses the inflammatory cytokines from macrophages [46]. Knockdown of 67LR impeded the anti-inflammatory effect of EGCG (1 μM) in mouse macrophages [5]. In addition, EGCG reduced TLR4 levels though 67LR-dependent mechanisms. It is known that overexpression of the Toll-interacting protein (Tollip) inhibits the activation of nuclear factor kappa B (NF-κB) and mitogen-activated protein kinases (MAPKs) in response to IL-1, TLR2, and TLR4 ligands [47]. Thus, Tollip negatively regulates the TLR-mediated signaling pathway. EGCG rapidly increased the level of Tollip and this effect is neutralized by 67LR knockdown. Therefore, 67LR is essential in manifesting the anti-inflammatory effect of EGCG and its ability to upregulate Tollip expression [48] (Figure 4). Peptidoglycan (PGN) is one of the pathogen-associated components derived from Gram-positive bacteria and strongly activates TLR2 signaling, whereas EGCG inhibits its effect by a mechanism involving 67LR and Tollip [48].

Furthermore, EGCG (2.5 μM) increased Tollip expression by downregulation of E74-like ETS factor 1 (Elf-1) [49] via PP2A/cGMP-dependent mechanisms. These results were further confirmed by in vivo studies: oral administration of EGCG and induction of cGMP, elevated expression of Tollip, upregulation of cGMP levels in macrophages, and downregulated Elf-1 expression. cGMP acted as a key molecule in 67LR-dependent upregulation of the E3 ubiquitin-protein ring finger protein 216 (RNF216) (Figure 4). Additionally, we demonstrated that EGCG downregulated TLR4 levels by upregulating RNF216 [6].

EGCG and the cGMP inducer are attractive negative regulators capable of attenuating TLR4 signaling. Aberrant activation of TLR4 plays an essential role in inflammation caused by obesity, which is involved in several disorders (e.g., hypertriglyceridemia, hyperinsulinemia, and cardiovascular disease). Moreover, TLR4 expression in adipose tissues is reduced by a highly absorbable 67LR agonist, (−)-epigallocatechin-3-*O*-(3-*O*-methyl)-gallate (EGCG3″Me) at the concentration of 1 μM. It completely inhibits obesity-elicited overexpression of inflammatory cytokines, such as monocyte chemoattractant protein-1 and tumor necrosis factor-α. The intake of EGCG3″Me inhibited obesity-induced hyperinsulinemia and hypertriglyceridemia. Collectively, 67LR is a potent target for relieving inflammation caused by obesity.

Mast cells and basophils mediate immunoglobulin (Ig) E-induced immediate allergic reactions. Allergen binding to IgE bound to the high-affinity IgE receptor (FcεRI) on the surface of mast cells or basophils leads to the release of preformed and newly generated inflammatory mediators, including histamine. Such mediators are responsible for the various symptoms of allergic reactions, such as atopic dermatitis, bronchial asthma, and food allergy [50,51]. The early phase of mast cell and basophil activation includes activation of protein tyrosine kinases and their substrates, an increase in the concentration of secondary messengers such as inositol trisphosphate and DAG, and elevation of intracellular Ca^2+^ levels [52,53]. The late phase of activation is caused after the influx of Ca^2+^ and includes the fusion of secretory granules with the cell membrane, remodeling of the actin cytoskeleton, and radical morphological changes [54,55,56]. EGCG inhibited calcium ionophore A23187-induced histamine release from human basophilic KU812 cells, but could not inhibit the elevation of intracellular Ca^2+^ levels after stimulation with A23187 [57]. Therefore, the effect of EGCG on histamine release will occur after the intracellular Ca^2+^ concentration has increased. Phosphorylation of MRLC^Thr18/Ser19^ temporally correlates with degranulation in rat basophilic RBL-2H3 cells, while its inhibition impairs degranulation [58]. EGCG, but not EGC, inhibits histamine release and reduces the concentration of phosphorylated MRLC [57]. The effect of EGCG (25 μM) on histamine release or the phosphorylation of MRLC^Thr18/Ser19^ was inhibited by treating with anti-67LR antibody or downregulating 67LR expression [57]. These results suggest that the inhibitory effect of EGCG on degranulation is induced by modifying the myosin cytoskeleton through the binding of EGCG to the cell surface 67LR. When basophilic cells were stimulated with A23187 in the presence of EGCG (25 μM), membrane ruffling, one effect of actin remodeling, was inhibited and biased F-actin accumulation was caused. Moreover, EGCG-induced actin remodeling was abrogated in both anti-67LR antibody-treated cells and 67LR-ablated cells [57]. These results indicate that EGCG-induced actin remodeling is caused by a reduction in MRLC ^Thr18/Ser19^ phosphorylation, mediated by the binding of EGCG to 67LR. Therefore, cytoskeletal remodeling may play an essential role in the inhibitory effect of EGCG on histamine release.

FcεRI is involved in the induction and maintenance of IgE-mediated allergic responses. In FcεRIα chain-deficient mice, IgE cannot bind to the surface of mast cells, thereby inhibiting the induction of degranulation [59]. Thus, downregulation of FcεRI expression in mast cells and basophils may contribute to attenuation of IgE-mediated allergic symptoms. We reported that EGCG (25 μM) can decrease the cell surface expression of FcεRI in KU812 cells, by inhibiting the phosphorylation of extracellular signal-regulated kinase1/2 (ERK1/2) [19]. Furthermore, the disruption of lipid rafts prevented the inhibitory effect of EGCG on ERK1/2. Thus, the interaction between EGCG and lipid rafts is important for the ability of EGCG to suppress FcεRI expression, and ERK1/2 may contribute to this suppression signal. We also revealed that the knockdown of 67LR inhibited the effect of EGCG on FcεRI expression. Moreover, the inhibitory effect of EGCG on the phosphorylation of ERK1/2 was abrogated in 67LR-ablated cells. Our findings indicate that the effect of EGCG on ERK1/2 phosphorylation correlates with 67LR expression, which implies that 67LR is the molecule responsible for the transduction of EGCG’s downregulatory signaling on FcεRI (Figure 4).

The *O*-methylated derivatives of EGCG, EGCG3″Me, and (−)-epigallpcatechin-3-*O*-(4-*O*-methyl)-gallate (EGCG4″Me), which were isolated from tea leaves such as Tong-ting oolong or Benifuuki tea cultivars, inhibit allergic reactions in vitro [60,61]. The in vivo anti-allergic effects of *O*-methylated EGCG, assessed using mouse models of type I and IV allergic reactions, were stronger than those of EGCG [60,62]. Mast cell activation was strongly inhibited by these methylated catechins, mediated through preventing tyrosine phosphorylation of cellular proteins, histamine/leukotriene release, and IL-2 secretion after FcεRI cross-linking [63]. A double-blind clinical trial assessed the efficacy of EGCG3″Me-rich Benifuuki green tea on allergic cedar pollinosis. Drinking 1.5 g of tea powder with water twice a day for 13 weeks improved the symptomatology of patients [64]. EGCG3″Me (25 μM) inhibited the release of histamine and suppressed the expression of FcεRI in KU812 cells, similar to EGCG [61,65]. The activity of EGCG3″Me was decreased by RNAi-mediated knockdown of 67LR expression [66]. Therefore, the anti-histaminic effect of EGCG3″Me is mediated by suppression of MRLC phosphorylation following 67LR binding. 67LR also mediates the suppressive effect of EGCG3″Me on FcεRI expression by reducing ERK1/2 phosphorylation.

EGCG is not stable and easily degraded in vivo. On the other hand, EGCG3″Me and EGCG4″Me are absorbed efficiently and are more stable than EGCG in animal and human plasma. This could explain the potent in vivo anti-allergic activities of *O*-methylated EGCG. EGCG undergoes methylation, and (−)-4′-*O*-methyl-epigallocatechin-3-*O*-(4-*O*-methyl) gallate (EGCG4′4″diMe) is a major metabolite of EGCG detected in plasma [67]. It did not demonstrate a suppressive effect on KU812 cells at the concentration of 25 μM [68]. Our findings of *O*-methylated EGCGs may lead to the understanding of the physiological activities of EGCG in vivo, but further study on the relationship between EGCG metabolites and 67LR is required.

### 2.5. MicroRNA-Mediated Anti-Cancer Effect of EGCG via 67LR

MicroRNAs (miRNAs) are small single-stranded, non-coding RNAs that regulate gene expression by inducing mRNA degradation or translational inhibition [69]. They play critical roles in various biological events, including cell proliferation [70], apoptosis [71], differentiation [72], inflammation [73], and metabolism [74], and are implicated in various diseases [75,76]. Some miRNAs are aberrantly expressed in various types of cancer cells and their dysregulation leads to cancer progression [77,78,79]. Therefore, modulating miRNA activity by dietary polyphenols may be an effective strategy against cancer [80,81,82,83]. Among the diverse polyphenols, some studies have investigated whether flavonoids similar to EGCG, belonging to flavan-3-ols, have such miRNA-mediated effects.

Isoflavones are a group of flavonoids that are found predominantly in soy and soy products. Their effects are mediated by estrogen receptors (ER). Equol is an intestinal metabolite of the representative soy isoflavone daidzein. At the concentration of 10 μM, it inhibits the proliferation of human cervical cancer HeLa cells and mouse melanoma B16 cells in an ER-independent manner. Its target may be the non-canonical poly(A) polymerase, PAP-associated domain containing 5 (PAPD5) [84]. Thus, oral administration of equol suppressed tumor growth in control mice inoculated with B16 cells, but not in mice inoculated with PAPD5-ablated B16 cells. Furthermore, it promoted tumor growth in PAPD5-ablated human breast cancer MCF-7 cells with high expression of ERα and induced polyadenylation of snoRNAs in a PAPD5-dependent manner. The expression of miR-320a in tumors was upregulated by its oral administration. Consequently, equol may show a dual effect on ER-positive cancer cells: it inhibits proliferation, an effect mediated by PAPD5 via miR-320a and enhances proliferation, an effect mediated by ERα.

The effects of delphinidin, a flavonoid belonging to anthocyanidins, on the levels of muscle-specific RING-finger protein 1 (MuRF1), miR-23a, and NFATc3 were assessed by in vitro and in vivo studies [85]. Delphinidin (5 μM) inhibits dexamethasone-induced upregulation of MuRF1 expression and downregulation of miR-23a and NFATc3 in mouse myoblast C2C12 cells. Its oral administration prevented the reduction of muscle mass in the gastrocnemius muscle, while reducing MuRF1 and increasing miR-23a and NFATc3 expression. This mechanism of action indicates that delphinidin intake may exert a preventive effect on disuse muscle atrophy.

Modulating miRNAs by flavonoids might be involved in their pharmacological properties. However, the effects of EGCG on miRNA expression in melanoma cells remain unclear. To address this, we conducted an miRNA microarray analysis [86]. EGCG (10 μM) upregulated the expression of miRNA-let-7b in melanoma cells by activation of the 67LR-dependent cAMP/PKA/PP2A signaling pathway. EGCG-induced upregulation of let-7b downregulated high-mobility group A2 (HMGA2), a target gene related to tumor progression. These observations offer a new insight on the mechanisms for EGCG-induced regulation of miRNA. The fact that EGCG can regulate miRNA via 67LR will provide a deeper understanding of the 67LR-mediated EGCG-sensing systems.

## 3. Potentiation of EGCG Activity by Modulating the 67LR-Dependent EGCG-Sensing Pathways

### 3.1. Anti-Cancer Effects of EGCG Are Potentiated by Modulation of Several EGCG-Sensing-Related Molecules

**Figure 5:**
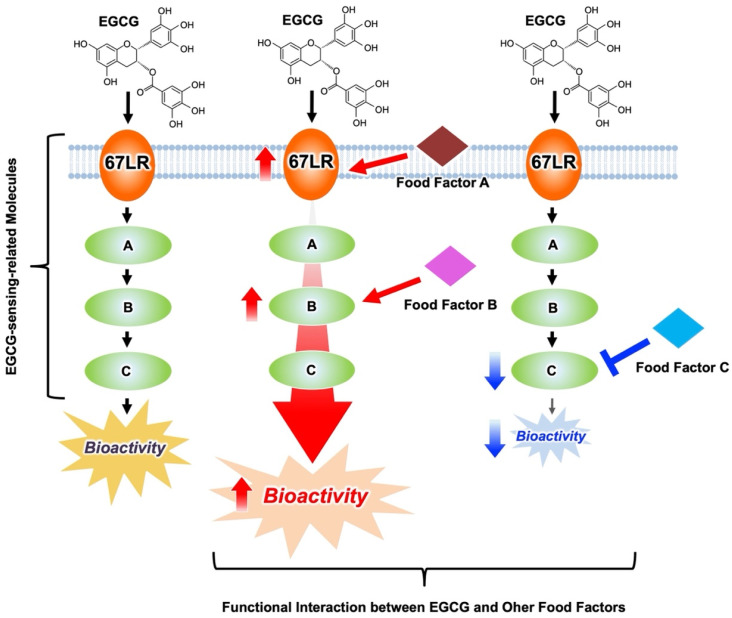
Regulation of EGCG bioactivity through modulating the behaviors of EGCG-sensing-related molecules by other food factors. Functional interaction between food factors, functional food pairing, includes both the increase and decrease in the activity and expression of EGCG-sensing-related molecules.

To assess the anti-cancer effects of an usual dietary intake of EGCG, it is essential to understand the combined effects of EGCG with other food ingredients that may potentiate its anti-cancer activity. As we have discussed, it is possible to effectively regulate the bioactivity of EGCG by modulating the activity or expression of molecules on the EGCG signaling pathways, i.e., EGCG-sensing-related molecules (Figure 5). If food factors can enhance the activity or expression of such EGCG-sensing-related molecules, they can present a useful combined effect with EGCG. Conversely, if the food factors weaken the activity or expression of such EGCG-sensing-related molecules, we can understand them to be an unfavorable combination with EGCG. Understanding such functional interactions between EGCG and other food factors, i.e., functional food pairings, could be a new approach to effectively utilize the diverse bioactivities of EGCG, including its anti-cancer effects.

As mentioned before, the expression of 67LR on the surface of B16 cells is enhanced by ATRA, an active derivative of vitamin A at the concentration of 0.1 μM. Therefore, ATRA can increase the binding of EGCG to the cell surface. EGCG (0.5 μM) and ATRA (0.1 μM) inhibited the growth of cells treated with a control antibody [2], while no such effect was observed following a pretreatment with an anti-67LR antibody. To investigate the in vivo anti-cancer effect of the combination treatment, mice were implanted with B16 cells and then treated with EGCG and/or ATRA. The combination substantially reduced the tumor volume compared to the vehicle. Oral administration of ATRA as monotherapy, or in combination with EGCG, increased the levels of 67LR expression in the tumor compared to the vehicle. After binding RAR, ATRA forms a heterodimer with retinoid X receptors (RXRs) for regulating the expression of specific genes. ATRA-induced enhancement of 67LR expression is reduced by knockdown of RAR [2]. The protein and cell surface levels of 67LR were enhanced by the aromatic retinoid (TTNPB), a pan-RAR agonist. Furthermore, the EGCG-induced inhibition of cell growth was enhanced upon treatment with TTNPB. Taken together, these findings suggest that the anti-tumor activity of EGCG may be enhanced by any compounds that activate RAR.

In addition to food factors, information on microenvironmental factors regulating 67LR expression is necessary for understanding the 67LR-mediated EGCG-sensing system. Hypoxia impacts progression of cancer and response to therapy. In cancer cells, the expression of many genes responsible for resistance to chemotherapy is increased by activating hypoxia-inducible factor 1 (HIF-1)-alpha. Exposure of various cancer cells to low O_2_ concentrations (5%) decreased the levels of 67LR protein expression and prevented EGCG (1 μM)-induced anti-cancer activity; however, the expression of HIF-1 was unaffected [87]. The downregulation of 67LR was prevented upon treatment with a proteasome inhibitor. This inhibitor restored sensitivity of cancer cells to EGCG even at low O_2_ concentrations (below 5%). In conclusion, the expression of 67LR is highly sensitive to O_2_ partial pressure. Consequently, the bioactivity of EGCG in cancer cells is regulated by O_2_ partial pressure. This fact is extremely important in understanding why there are differences in the effects of EGCG in cell culture systems and animal models/humans because the O_2_ partial pressure in cell culture systems (160 mmHg, 21%) is much higher than that in blood or tissues (<40 mmHg, 5%). It is assumed that the anti-cancer effects of EGCG are less effective in vivo than in vitro. Therefore, to manifest the anti-cancer effects of EGCG in vivo, it is important to enhance the 67LR expression as well as the activity and expression of 67LR-mediated EGCG-sensing-related molecules. It is reasonable to promote research on functional food pairing focusing on the 67LR-mediated EGCG-sensing system in order to manifest the in vivo physiological effects of EGCG.

cGMP plays an essential role in EGCG-induced MM-apoptosis. Physiologically achievable levels of EGCG can sufficiently increase NO production but not that of cGMP, essential for EGCG to induce MM apoptosis [4]. Therefore, the upregulation of cGMP production may be a “checkpoint” in the EGCG-elicited activation of the proapoptotic signaling pathway. cGMP is inactivated by phosphodiesterases (PDEs) with hydrolyzing of the 3,5′-phosphodiester bonds. Combination of EGCG (5 μM) with PDE5-selective inhibitors (5 μM) substantially inhibited cell proliferation [4]. The protein levels of both PDE5 and 67LR in MM cells are considerably higher than in normal PBMCs. Furthermore, levels of the two molecules are increased in several human cancers (breast, gastric, pancreatic, and prostate) compared to their normal counterparts. Vardenafil, a PDE5 inhibitor used for the treatment of erectile dysfunction, did not affect the number of viable normal PBMCs from healthy donors, but significantly potentiated the killing effect of EGCG on primary MM cells from patients. EGCG in combination with vardenafil inhibited the proliferation of the gastric cancer cell line MKN45, pancreatic cancer cell line PANC-1, prostate cancer cell line PC3, and AML cells, but did not affect normal human diploid fibroblasts or normal human umbilical vein endothelial cells [4]. To examine the in vivo effect of this combination, EGCG and/or vardenafil were intraperitoneally administered to female mice previously inoculated with MDA-MB-231 cells. Significant suppression of tumor growth was observed with the combination of EGCG and vardenafil.

### 3.2. Citrus Polyphenols Potentiate Bioactivites of EGCG

To identify the green tea extract compounds that potentiate EGCG activity, metabolic profiling of 43 tea cultivars was performed using liquid chromatography−mass spectrometry. Although EGCG alone (5 μM) showed no bioactivity, the flavanone eriodyctiol has been found to potentiate EGCG-induced apoptosis in MM cells (both compounds were used at a concentration of 5 μM), and in a mouse tumor model, by amplifying the activation of the 67LR/Akt/eNOS/PKCδ/ASM pathway elicited by EGCG [9]. This flavanone is one of the most abundant polyphenols in *Citrus limon* (lemon) [88] and an intermediate metabolite of the catechin synthesis pathway in *Camellia sinensis* (tea) [89]. Eriodictyol and its analogues, naringenin and hesperetin, enhanced the anti-proliferative activity of *O*-methylated EGCG (EGCG3″Me) (5 μM) on MM cells at the concentration of 5 μM.

Although EGCG alone (5 μM) showed no bioactivity, fustin, a flavanonol, enhanced EGCG (5 μM)-induced activation of the eNOS/cGMP axis (Src/Akt/eNOS/cGMP signaling pathway) in MM cells at the concentration of 5 μM [90]. This flavanonol is a structural isomer of eriodictyol and is found in young fustic (*Cotinus coggygria*) and lacquer trees (*Toxicodendron vernicifluum*) [91]. It synergically enhances the proapoptotic effect of EGCG on MM cells, without affecting normal cells. The in vivo combined effects of EGCG and fustin were assessed using a mouse model in which female BALB/c mice were inoculated with MM MPC11 cells. Consistent with the in vitro findings, this combination suppressed tumor growth in vivo, by increasing eNOS^Ser1177^ phosphorylation. The EGCG-elicited activation of the eNOS/cGMP signaling was amplified by Fustin. Furthermore, the combination of EGCG/fustin, while effective, did not influence alanine aminotransferase/aspartate aminotransferase levels, which is the dose-limiting toxicity of EGCG.

Basophilic degranulation is inhibited by EGCG3″Me mediated through 67LR. We investigated the mechanisms responsible for this inhibitory action of EGCG3″Me and that of EGCG3″Me and eriodictyol in combination [10]. This catechin inhibited the release of β-hexosaminidase from the rat basophilic/mast cell line RBL-2H3 induced by stimulation of IgE/antigen binding, and induced the activity of ASM. These effects were abrogated by pretreatment with anti-67LR antibody. Desipramine, the ASM-specific inhibitor, prevented the suppression of degranulation induced by EGCG3″Me. The sGC inhibitor NS2028 reduced the effect of EGCG3″Me while the sGC activator BAY41-2272 suppressed IgE/antigen-mediated degranulation. In addition, the effects of EGCG3″Me (0.5 μM) on the activity of ASM and degranulation were significantly potentiated by eriodictyol (0.1 μM). Moreover, the suppressive potency of EGCG3″Me-rich Benifuuki green tea on IgE/antigen-induced passive cutaneous anaphylaxis (PCA) in BALB/c mice was enhanced by oral administration of eriodyctiol-7-*O*-glucoside, derived from lemon peels. These observations indicate that EGCG3″Me inhibits IgE/antigen-mediated degranulation by activating the 67LR/sGC/ASM signaling pathway and that this signaling is amplified by eriodictyol.

Eriodictyol-7-*O*-glucoside did not show an anti-allergic effect after a single dosing, but significantly enhanced the anti-allergic effect of Benifuuki green tea at 2 mg/kg body weight (b.w.) (4.4 μmol/kg b.w.). An anti-allergic effect of eriodictyol (non-glycosylated form) has been found after a single dosing [92]. The IgE/antigen-induced PCA reaction in BALB/c mice was inhibited by the oral administration of doses of eriodictyol over 30 mg/kg b.w. (104.1 μmol/kg b.w.), but not that of doses lower than 10 mg/kg b.w. (34.7 μmol/kg b.w.). Thus, a combination therapy with eriodictyol could be effectively used for preventing IgE/antigen-induced allergic reaction even at less than 1/20 (4.4/104.1 μmol) dosing. These results suggest that combining different foods/food components, which are inactive when ingested separately, may have potential for the design of new anti-allergic foods and their combinations (e.g., green tea and lemon peels), and for therapeutic application of medicinal herbs and botanical extracts to prevent IgE-mediated immediate hypersensitivity.

Obesity is induced by typical Western diets, which are associated with a high risk of dyslipidemia. Recently, we examined the combined potency of green tea and eriodictyol in a mouse model using a Western diet. C57BL/6J mice were fed a normal diet, high-fat and high-sucrose diet (HF/HS), HF/HS with green tea extract diet (HF/HS + GT), HF/HS with eriodictyol diet (HF/HS + Eri), or HF/HS with green tea extract and eriodictyol diet (HF/HS + GT + Eri) [7]. The lowering of body weight was observed in the HF/HS + GT + Eri group compared with the HF/HS group. The HF/HS diet induced the elevation of total cholesterol and low-density lipoprotein (LDL) levels, the hepatic mRNA expression of 3-hydroxy-3-methylglutaryl-coenzyme A (HMG-CoA) reductase (HMG-CR), and that of HMG-CoA synthase (HMG-CS). These changes were significantly prevented by the addition of both green tea extract and eriodictyol to the diet. Furthermore, LDL receptor (LDLR) levels were higher in the HF/HS + GT + Eri group than in the HF/HS group. These findings suggest that cholesterol levels are decreased by combining green tea with eriodictyol, mediated through inhibiting HMG-CR and HMG-CS and upregulating LDLR levels in the liver.

Therefore, bioactivities of EGCG are potentiated by intake of Citrus polyphenols simultaneously. Nevertheless, the effects of this combination have scarcely been verified in human trials. Recently, we examined the anti-obesity effect of a combination of EGCG and α-glucosyl hesperidin (GT-gH), a derivative of citrus polyphenol, using doses that were not associated with anti-obesity effects when the substances were evaluated separately in clinical trials [12]. α-Glucosyl hesperidin, a water-soluble hesperidin derivative used as a food additive, is considerably more water-soluble than hesperidin. This hesperidin derivative is converted into hesperetin in the gut and can be further metabolized to eriodictyol in the liver and kidney [93]. Therefore, in vivo α-glucosyl hesperidin should exert effects similar to hesperetin or eriodictyol. In this randomized, placebo-controlled, double-blind, clinical trial with parallel design, 60 healthy Japanese men and women aged 30–75 years consumed green tea combined with α-glucosyl hesperidin (GT-gH), corresponding to 178 mg/day α-glucosyl hesperidin and 146 mg/day EGCG, for 12 weeks [12]. Physical, hematological, blood biochemical, and urine examinations indicated that the use of GT-gH is safe. At week 12, the consumption of GT-gH suppressed weight gain and reduced body mass index (BMI) compared with placebo. This effect was more pronounced in patients aged <50 years: when compared to control, triglyceride and body fat percentage reduced at week 6, visceral fat level, body fat percentage, body weight, BMI, and blood LDL/HDL ratio reduced at week 12.

### 3.3. Sulfur-Containing Food Factor Potentiates Bioactivites of EGCG

We highlighted the importance of cGMP as a mediator of the EGCG-sensing system. Inhibition of the enzymatic activity of PDE5 and PDE3 is expected to potentiate EGCG action [4,41,94]. Food factors known to inhibit PDE5 include sulfur-containing compounds such as diallyl trisulfide (DATS) and diallyl disulfide (DADS), metabolites derived from components abundant in plants of the genus Allium, such as garlic, leek, and onion. They potentiated the anti-tumor activity of EGCG [95] and the suppressive potency of green tea extract on fat accumulation [96]. PDE5 protein levels in adipocytes (3T3-L1 cells) were found to be significantly elevated compared to those in preadipocytes. This elevation was significantly suppressed by DADS treatment, while EGCG alone showed a decreasing trend in PDE5 expression. The combination of EGCG (5 μM) and DADS (10 μM) increased the expression of genes involved in lipid metabolism. In the adipose tissues of mice, the HF/HS diet-induced upregulation of PDE5 expression was suppressed by DADS. The HF/HS diet-induced adipose tissue increase and triglyceride accumulation in the liver was attenuated by green tea extract/DADS combination, mediated through preventing the upregulation of fatty acid synthesis-related enzymes, such as sterol regulatory element-binding protein-1 (SREBP-1), fatty acid synthase, and stearoyl-CoA desaturase-1. Furthermore, this combination caused an upregulation of thermogenesis-related genes, such as peroxisome proliferator-activated receptor (PPAR) γ coactivator 1 α and uncoupling proteins, in both white and brown adipose tissues. Taken together, DADS potentiates the anti-obesity action of green tea extract, as it downregulates SREBP-1 expression levels and activates the PPAR axis. Using this combination might be a novel and easily applicable dietary approach for obesity-related diseases.

### 3.4. Fatty Acids Modulate Anti-Obesity Effect of Green Tea Extract

Diet-induced obesity was reduced by green tea and its major polyphenol, EGCG. However, it is not clearly understood how the type of diet influences the intensity of these anti-obesity effects (strong vs. weak). Recently, we investigated whether the composition of fat and fatty acids in food influences the anti-obesity potency of green tea extract in a mouse model using HF diet [97]. The intake of green tea extract drastically prevented weight gain and fat accumulation induced by an olive oil-based HF diet, but only slightly those induced by a beef tallow-based HF diet. Furthermore, green tea extract inhibits more strongly obesity induced by an unsaturated fatty acid-enriched HF diet, than that induced by a saturated fatty acid-enriched HF diet. The underlying cause of these differences might be upregulation of the expression of PPARδ signaling pathway-related genes induced by green tea extract in the white adipose tissue, and regulation of gene expression associated with the activation of the EGCG signaling pathway. We demonstrated that the anti-obesity effect of green tea extract differed depending on the type of fat or fatty acids in the diet and was reduced by the presence of saturated fatty acids. These differences could be caused by the modulation of the expression of the genes related to the EGCG signaling pathway. Therefore, drinking of green tea and replacement of saturated fatty acids, such as those contained in animal fat, with unsaturated fatty acids, such as those contained in olive oil and other vegetable oils, could represent useful strategies for preventing obesity.

## 4. Conclusions

**Figure 6:**
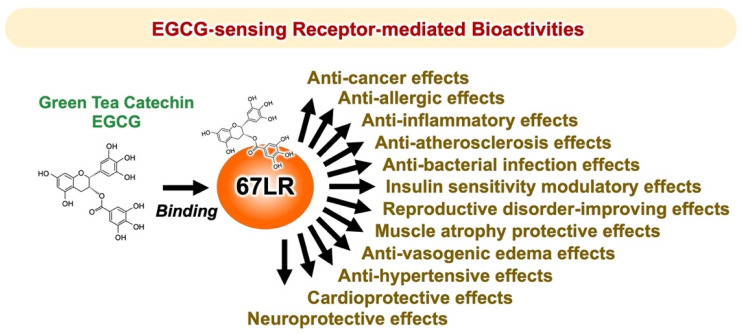
67LR is an important sensing molecule that responds to EGCG and mediates its various biological activities.

As we have discussed, 67LR is an important sensing molecule for EGCG and confers its beneficial properties, including anti-cancer [1,40,98,99,100], anti-atherosclerosis [101,102,103], modulation of insulin sensitivity [104,105,106,107], anti-bacterial [108], anti-inflammatory [5,48,109,110,111], anti-allergic [10,18,25,57,66], anti-hypertensive [112], anti-vasogenic edema [113,114], as well as being protective against muscle atrophy [8,115], reproductive disorders [116], and cardiovascular [117,118,119] and neurological diseases [120,121,122] (Figure 6). MYPT1, eEF1A, PP2A, cGMP, and ASM are endogenous EGCG-sensing-related molecules that are essential to manifest the in vivo protective effects of EGCG. Such factors mediate specific signaling pathways that are evoked by physiologically relevant concentrations of EGCG. They are “master factors,” which by enhancing the disease-preventive activity of EGCG may influence its use in clinical practice. Only diseases with high expression of these “master factors” might respond to physiologically relevant concentrations of EGCG, while low expression might result in “EGCG tolerance” in which the physiological effects of EGCG are not manifested.

In this review, functional food pairing of green tea/EGCG with a citrus polyphenol α-glucosyl hesperidin, focusing on their anti-obesity effects, was demonstrated in a human intervention study, but data on other pairing effects are limited to in vitro and animal studies. Therefore, if several functional food pairings described here are demonstrated in humans, the importance of the concept of food factor-sensing system will be confirmed, and development of functional food, dietary design, and drug discovery based on new strategies focusing on the EGCG-sensing system will be expected.

**Figure 7:**
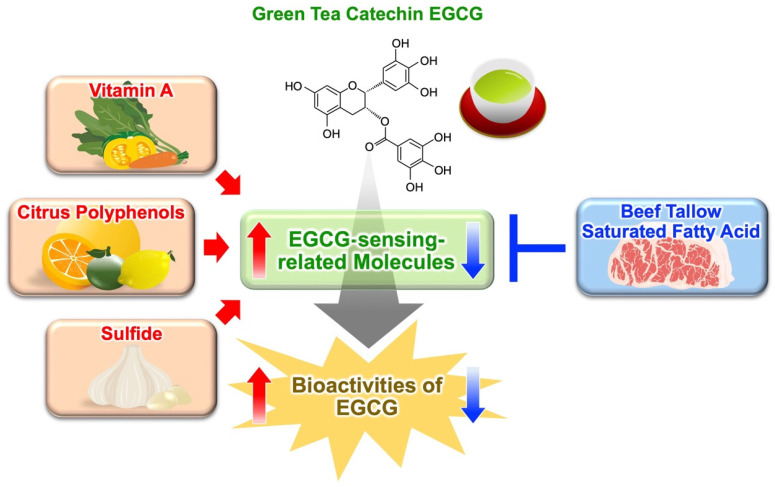
Functional food pairing of EGCG with food factors capable of enhancing or weakening the activity of EGCG-sensing system.

The intensity of the functional effect of a food factor is believed to be determined by the amount of food consumed. However, as shown in this review, the bioactivities of EGCG and green tea extract are greatly influenced by the food factors consumed simultaneously, such as vitamins, sulfur-containing compounds, citrus-derived components, and various oils and fats (Figure 7). Green tea is one of the foods that characterize the Japanese diet. Although it is known that the Japanese diet contains a wide variety of food factors, the contribution of their combination to the functionality of this diet is largely unknown. We hope that understanding functional food pairing and the interactions between food factors will offer scientific evidence on the health benefits of the Japanese diet. Furthermore, Chinese herbal medicines exert their pharmacological effects by combining multiple herbal medicines—so a combination of EGCG and a food factor that enhances EGCG sensing may be seen in a similar manner as the Chinese herbal medicine and prescribed on a scientific basis.

Recently, various target molecules of EGCG for deciphering the molecular mechanisms underlying its physiological effects have been identified [15,16]. However, the relationships among those identified target molecules are often not known, and their relevance to 67LR is not at all clear. Understanding the crosstalk between the currently proposed mechanisms will lead to a more precise mechanism of EGCG action and expand the potential of the 67LR-mediated EGCG-sensing system. We have successfully generated 67LR knockout mice and are now analyzing the function of 67LR. Although there are currently no useful reported cases, it is hoped that molecular docking studies will elucidate the detailed interactions between 67LR and EGCG. Further functional analysis of other EGCG-sensing-related molecules will allow us to propose more effective ways to utilize EGCG and to search for food factors/drugs that mimic EGCG action, and provide optimal molecular targets for finding EGCG-based functional food pairing and understanding EGCG sensitivity.

The metabolism of EGCG after intake is important for its action in the human body and affects its clinical applications. Green tea catechins are converted to catechin metabolites in the intestine and liver. Recently, we have shown that intestinal metabolites of green tea catechin enhance CD4^+^ T cell activity and natural killer cell activity [123]. In particular, 5-(3′,5′-dihydroxyphenyl)-γ-valerolactone (EGC-M5), the major metabolite of EGCG, showed much stronger activity than EGCG. However, it is unclear how the 67LR-mediated EGCG-sensing system is involved in the physiological effects of EGCG metabolites, including EGC-M5. In our body, the enteric barrier works to prevent high concentrations of polyphenols in blood, and in some cases, metabolites of polyphenols arrive in plasma and achieve concentrations generating physiological effects. Currently, it is not clear to what extent intact EGCG and its metabolites contribute to the physiological effects of EGCG. Elucidating the sensing mechanisms of EGCG metabolites will be essential to reconsider the physiological significance of intact EGCG at physiologically relevant concentrations and to demonstrate EGCG-based functional food pairing at the human level.

Currently, no clinical trials have established the direct relationship between 67LR and EGCG. Cohort studies and human interventional trials will provide more definitive information on the potential relationship between EGCG-sensing pathways and health-promoting properties of EGCG intake. We expect that this review will have broad implications that may be applicable to research on other dietary components.

## Data Availability

Not applicable.

## Sample Availability

Samples of the compounds are not available from the authors.

## Abbreviations

67LR	67-kDa laminin receptor
AC	adenylate cyclase
AML	acute myeloid leukemia
ASM	acid sphingomyelinase
ATRA	all-trans-retinoic acid
BMI	body mass index
b.w.	body weight
C	(−)-catechin
CG	(−)-catechin-3-*O*-gallate
C_max_	maximum plasma concentration
CPI17	C-kinase potentiated protein phosphatase-1 inhibitor protein of 17-kDa
DADS	diallyl disulfide
DAG	diacylglycerol
DATS	diallyl trisulfide
EC	(−)-epicatechin
ECG	(−)-epicatechin-3-*O*-gallate
eEF1A	eukaryotic elongation factor 1A
EGC	(−)-epigallocatechin
EGCG	(−)-epigallocatechin-3-*O*-gallate
EGCG3″Me	(−)-epigallocatechin-3-*O*-(3-*O*-methyl) gallate
EGCG4″Me	(−)-epigallpcatechin-3-*O*-(4-*O*-methyl) gallate
EGCG4′4″diMe	(−)-4′-*O*-methyl-epigallocatechin-3-*O*-(4-*O*-methyl) gallate
Elf-1	E74-like ETS factor 1
eNOS	endothelial nitric oxide synthase
ER	estrogen receptor
ERK1/2	extracellular signal-regulated kinase1/2
FAK	focal adhesion kinase
FcεRI	high-affinity IgE receptor
GCG	(−)-gallocatechin-3-*O*-gallate
GT-gH	green tea combined with α-glucosyl hesperidin
HDL	high-density lipoprotein
HF/HS	high-fat and high-sucrose diet
HF/HS + Eri	HF/HS with eriodictyol diet
HF/HS + GT	HF/HS with green tea extract diet
HF/HS + GT + Eri	HF/HS with green tea extract and eriodictyol diet
HIF-1	hypoxia-inducible factor 1
HMG-CoA	3-hydroxy-3-methylglutaryl-coenzyme A
HMG-CR	HMG-CoA reductase
HMG-CS	HMG-CoA synthase
HMGA2	high-mobility group A2
Ig	immunoglobulin
IL	interleukin
IL-4R	IL-4 receptor
*K_d_*	dissociation constant
LDL	low-density lipoprotein
LDLR	LDL receptor
LPS	lipopolysaccharide
MAPKs	mitogen-activated protein kinases
miRNA/miR	microRNA
mTOR	mammalian target of rapamycin
MM	multiple myeloma
MRLC	myosin regulatory light chain
mTOR	mammalian target of rapamycin
MuRF1	muscle-specific RING-finger protein 1
MYPT1	myosin phosphatase target subunit 1
NF-κB	nuclear factor kappa B
p70S6K	p70S6 kinase
PAPD5	PAP-associated domain containing 5
PB1	procyanidin B1
PB2	procyanidin B2
PBMCs	peripheral blood mononuclear cells
PC1	procyanidin C1
PCA	passive cutaneous anaphylaxis
PDEs	phosphodiesterases
PGN	peptidoglycan
PKA	protein kinase A
PKCδ	protein kinase Cδ
PLC	phospholipase C
PP2A	protein phosphatase 2A
PPAR	peroxisome proliferator-activated receptor
RAR	retinoic acid receptor
RNF216	E3 ubiquitin-protein ring finger protein 216
RXRs	retinoid X receptors
SET	Suvar3–9, enhancer-of-zeste, trithorax
sGC	soluble guanylate cyclase
shRNA	short hairpin RNA
SREBP-1	sterol regulatory element-binding protein-1
TLR4	Toll-like receptor 4
Tollip	Toll-interacting protein
